# Early bone-formation response to sclerostin antibody is altered by prior antiresorptive treatment in ovariectomized rats

**DOI:** 10.1093/jbmrpl/ziag115

**Published:** 2026-07-21

**Authors:** Agathe Bédard, Denise Dwyer, Melanie Felx, Martin Guillot, Amy Bourdeau, Marina Stolina, Jen Timoshanko, Zhigang Yu, Mary Oates, Rogely Waite Boyce, Serge Ferrari

**Affiliations:** Charles River Laboratories Montreal ULC, Senneville, QC H9X 3R3, Canada; Department of Cardiometabolic Disorders, Amgen Inc., Thousand Oaks, CA 91320, United States; Charles River Laboratories Montreal ULC, Senneville, QC H9X 3R3, Canada; Charles River Laboratories Montreal ULC, Senneville, QC H9X 3R3, Canada; Department of Translational Safety and Bioanalytical Sciences, Amgen Inc., Thousand Oaks, CA 91320, United States; Department of Cardiometabolic Disorders, Amgen Inc., Thousand Oaks, CA 91320, United States; UCB, Slough SL1 3WE, United Kingdom; Department of Global Development, Amgen Inc., Thousand Oaks, CA 91320, United States; Department of Global Development, Amgen Inc., Thousand Oaks, CA 91320, United States; Department of Translational Safety and Bioanalytical Sciences, Amgen Inc., Thousand Oaks, CA 91320, United States; Geneva University Hospital, 1205 Geneva, Switzerland

**Keywords:** denosumab, OPG-Fc, romosozumab, sclerostin antibody, treatment sequence

## Abstract

To explore how preceding treatment with a bisphosphonate (BP) and/or OPG-Fc, a RANKL inhibitor, affects early bone formation (BF) response to sclerostin antibody (Scl-Ab) in vertebral cancellous and cortical bone, we treated ovariectomized rats with various sequences of vehicle (Veh), OPG-Fc, and alendronate (Aln) or zoledronic acid (ZOL) in cycle 1 (0-12 wk) or cycle 2 (12-18 wk) before administering Scl-Ab in cycle 3 (18-24 wk). Treatment sequences included Veh/Veh/Scl-Ab (treatment naïve), OPG-Fc/OPG-Fc/Scl-Ab, OPG-Fc/ZOL/Scl-Ab, Aln/OPG-Fc/Scl-Ab, and OPG-Fc/OPG-Fc_Veh/Scl-Ab (4-wk treatment-free period preceding Scl-Ab). DXA scans and serum bone turnover markers were assessed. Cancellous and endocortical histomorphometric endpoints were quantified at weeks 18, 20, and 24. With Veh/Veh/Scl-Ab, anticipated bone responses to Scl-Ab were observed. From week 18 to week 24, Scl-Ab-mediated bone mass accrual was blunted with preceding antiresorptive sequences. In groups where OPG-Fc immediately preceded Scl-Ab, BF suppression was marked at week 20 with limited modeling-based bone formation (MBBF). In contrast, when ZOL was interposed between OPG-Fc and Scl-Ab, MBBF was modestly reduced at week 20. By week 24, BF surfaces were generally similar in all groups; the contribution of MBBF and remodeling-based bone formation (RBBF) differed. In contrast to other antiresorptive sequences, in OPG-Fc/OPG-Fc_Veh/Scl-Ab, RBBF was also a significant contributor to bone forming surfaces, reflecting a rebound in resorption and remodeling. With the other antiresorptive sequences, MBBF was the major contributor to BF, consistent with prolonged suppression of remodeling at week 24. All prior antiresorptive sequences blunted Scl-Ab effects on early BF. BP exposure (before or after OPG-Fc) improved cumulative effects across all cycles on bone mass compared with OPG-Fc alone. Interposing ZOL between OPG-Fc and Scl-Ab improved the early BF response to Scl-Ab.

## Introduction

Randomized controlled trials have underscored the importance of the treatment sequence of romosozumab with antiresorptive agents. Results from these trials demonstrated that previous antiresorptive therapies can impact the response to romosozumab in terms of effects on bone mass and bone turnover markers (BTMs).^1–6^ In the FRAME and ARCH trials, where romosozumab was administered for 12 mo as initial therapy, BMD gains in both trials were ~6% at the TH and >13% at the LS.^2^^,^^3^ These gains in bone mass were associated with dynamic changes in the bone formation marker N-terminal propeptide of type I collagen (P1NP) with an early robust increase at 1 mo (median % increase from baseline ~150% FRAME ~80% ARCH) that attenuated over the 12-mo treatment period accompanied by a sustained decrease in the bone resorption marker C-terminal telopeptide of type I collagen (CTx). In contrast, in the STRUCTURE trial,^4^ where romosozumab was administered for 12 mo following at least 36 mo of bisphosphonates (BPs) and 12 mo of alendronate before screening, BMD response to romosozumab treatment was reduced at the TH (2.9%) and LS (9.8%) compared with the FRAME and ARCH trials.^2^^,^^3^ in spite of ~150% increase in P1NP at 1 mo. In the romosozumab phase 2 extension trial,^5^ prior treatment of naïve patients with denosumab (a RANKL inhibitor that is an antiresorptive agent with a different mechanism of action than that of BPs) for 12 mo followed by romosozumab for 12 mo resulted in an even more diminished increase in BMD at the TH (0.9%) and LS (5.3%). Notably, the P1NP and CTx profiles in response to romosozumab treatment differed from those observed in the FRAME, ARCH, and STRUCTURE trials.^2–4^ P1NP and CTx were significantly decreased at the end of denosumab treatment, and when patients directly transitioned to romosozumab, the increase in P1NP at 1 mo was blunted (~35% compared to ~50% in treatment-naïve subjects) followed by a gradual increase in P1NP and CTx to or above baseline.^5^ In another Phase II extension trial,^6^ subjects received 24 mo of romosozumab followed by 12 mo of denosumab or placebo then transitioned to a second 12 mo course of romosozumab. BMD gains in response to the second course of romosozumab were significantly lower in subjects directly transitioned from denosumab. The increase in P1NP at 1 mo was blunted (~10% compared to ~100% in subjects transitioning from placebo) followed by a gradual increase in P1NP and CTx to above baseline. Similar differential effects of prior treatment with BPs or denosumab on the BTM and bone mass increases in response to romosozumab have also been reported in prospective observational studies, where early changes in BTM predicted the subsequent effects on bone mass.^7–9^

Changes in BTMs in response to romosozumab reflect global effects on bone formation and resorption at the bone tissue level. To date, FRAME is the only romosozumab clinical trial with a bone biopsy sub-study for the investigation of tissue-level effects on bone formation and resorption.^10^ Histomorphometric analyses revealed that romosozumab increased the dynamic parameters of bone formation on the endocortical (Ec) and cancellous (Cn) surfaces at month 2 of treatment associated with a decrease in resorption parameters, with no effect on periosteal or intracortical parameters.^10^ The increase in bone formation has been characterized as modeling-based bone formation (MBBF), with a minor contribution from a transient positive effect on wall thickness in remodeling sites.^11^^,^^12^ By month 12, bone formation with romosozumab was significantly decreased compared with placebo reflecting a decrease in bone turnover, but the decrease in resorption parameters was sustained, resulting in a positive bone balance at the remodeling unit.^11^ This could explain the continuous BMD gain up to 12 mo even when bone forming surfaces had decreased. These histomorphometric changes reflect the dynamic changes in BTMs observed in studies involving treatment-naïve patients.

The bone tissue-level changes in bone formation and resorption in response to romosozumab in patients who have received antiresorptive treatments prior to romosozumab are unknown. We conducted a study in mature ovariectomized rats to explore how preceding treatment with a BP (zoledronic acid [ZOL] or alendronate [Aln]) and/or OPG-Fc, a RANKL inhibitor, administered in various sequences, affects the subsequent early response to sclerostin antibody (Scl-Ab), including effects on BTMs, bone mass/density, and tissue-level bone formation on Cn and cortical bone in the lumbar vertebrae.

## Materials and methods

### Animals

A total of 150 female Sprague-Dawley rats (Hsd:Sprague Dawley SD; Envigo [Inotiv]) aged 6-6.5 mo were ovariectomized and maintained for an additional 8-9 wk to allow for bone depletion. Animals were cared for in accordance with the Guide for the Care and Use of Laboratory Animals, Eighth Edition.^13^ All research protocols were approved by the IACUC and all animal experiments complied with ARRIVE (Animal Research: Reporting of In Vivo Experiments) guidelines.

### Study design, dosing, and dose selection

**Figure 1:**
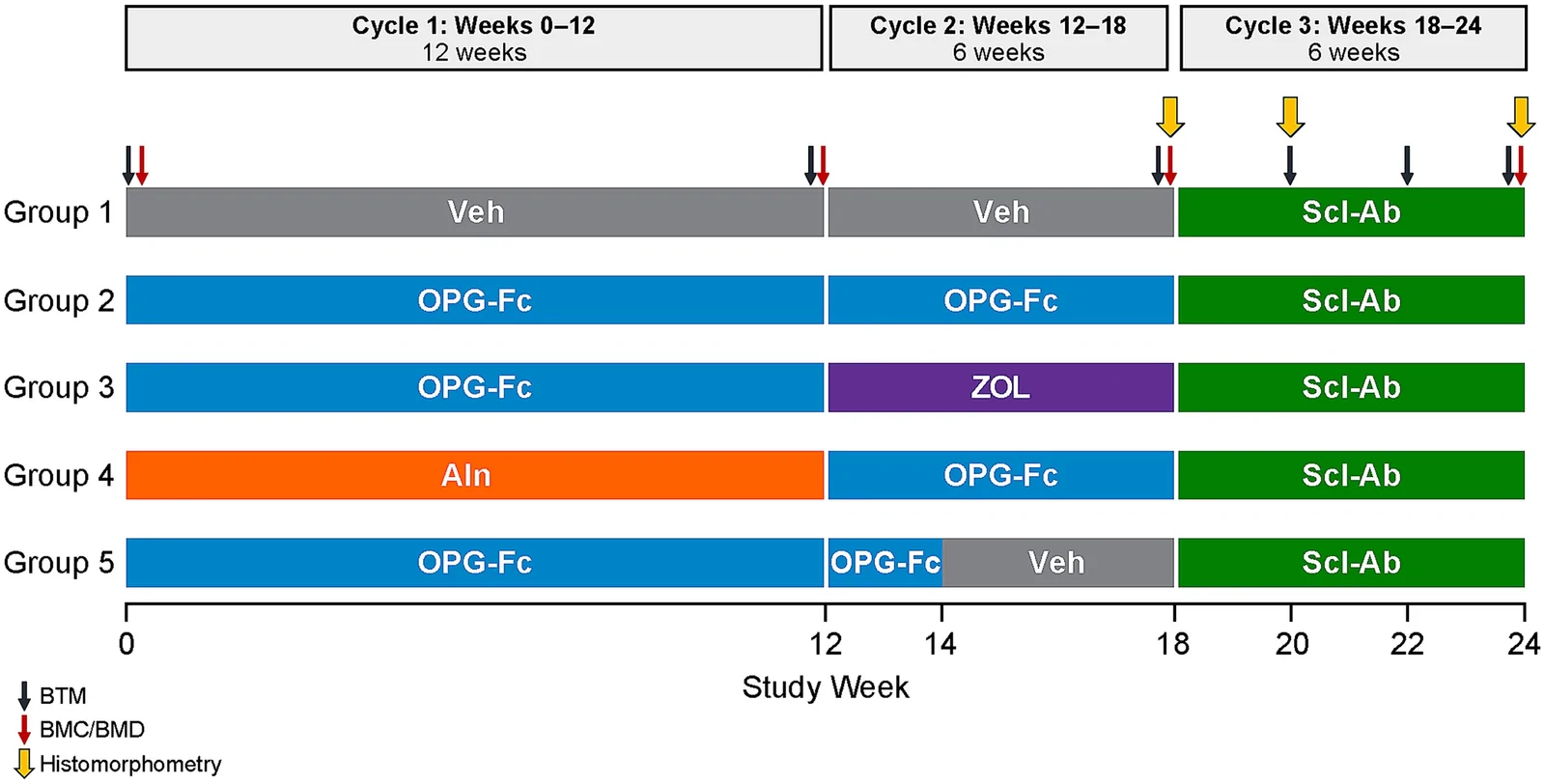
Study design to examine the effects of preceding antiresorptive treatment sequences on response to Scl-Ab. Group 1 was administered Veh in cycles 1 and 2, and then Scl-Ab in cycle 3 (Veh/Veh/Scl-Ab) to serve as the naïve response comparator. Treatment sequences were designed to model various treatment sequences that have been used in the clinical setting. To compare response to Scl-Ab when preceded by OPG-Fc, group 2 (OPG-Fc/OPG-Fc/Scl-Ab) and group 5 (OPG-Fc/OPG-Fc_Veh/Scl-Ab) were administered OPG-Fc in cycles 1 and 2. Group 5 (OPG-Fc/OPG-Fc_Veh/Scl-Ab) had an intervening 4-wk treatment-free period prior to the transition to Scl-Ab to determine if the response to Scl-Ab was different when OPG-Fc was clearing or cleared compared with direct transition from OPG-Fc. Group 3 (OPG-Fc/ZOL/Scl-Ab) was administered OPG-Fc in cycle 1 and ZOL in cycle 2 to determine if interposed ZOL treatment between OPG-Fc and Scl-Ab affects response to Scl-Ab. Group 4 (Aln/OPG-Fc/Scl-Ab) was administered Aln in cycle 1 and OPG-Fc in cycle 2 to determine if initial treatment with BP followed by OPG-Fc affected response to Scl-Ab, and if the preceding BP treatment blunted the expected rise in resorption markers as OPG-Fc was clearing in cycle 3. Aln, alendronate; BP, bisphosphonate; BTM, bone turnover marker; Scl-Ab, sclerostin antibody; Veh, vehicle; ZOL, zoledronic acid.

Rats were randomized into 5 treatment groups (30 rats/group) as illustrated in Figure 1, controlled by predose spinal BMD to assure group homogeneity. Treatment cycle length was 12 wk in cycle 1 and 6 wk in both cycles 2 and 3.

All treatments were administered at 0.5 mL/kg s.c., except ZOL, which was administered i.v. at 1 mL/kg in the tail vein. The vehicle (Veh) for Scl-Ab was 10 mM sodium acetate, 9.0% sucrose, and 0.004% polysorbate 20 (pH 5.0). Veh for OPG-Fc was PBS (1× PBS, pH 7.4). Veh for ZOL and Aln was 0.9% sodium chloride, USP.

A dose of 5 mg/kg/wk was chosen for Scl-Ab as this dose is expected to provide exposure equivalent to the human clinical exposure at 210 mg monthly romosozumab based on area under the curve. OPG-Fc was administered at 10 mg/kg twice weekly; this dose completely blocks bone resorption and osteoclastogenesis and upon discontinuation for a 4-wk period is sufficiently cleared to allow bone resorption and osteoclastogenesis to resume in mice.^14^ ZOL was administered as a single i.v. dose of 0.1 mg/kg, a dose that provides protection from bone loss in ovariectomized rats for up to 32 wk.^15^ Aln was administered at a dose of 0.035 mg/kg twice weekly. This Aln dose is expected to reduce mineralizing surface (MS/BS) by approximately 70%-80% based on previous studies in ovariectomized rats at a slightly lower dose.^16^^,^^17^

### BTM assessments

Serum samples were collected from all rats prior to dosing and at weeks 12, 18, 20, 22, and 24 for the analysis of osteoclast-derived tartrate-resistant acid phosphatase form 5b (TRAcP 5b), CTx, and P1NP (Supplementary Methods).

### In vivo densitometry

Densitometry scans and assessments were performed using DXA (Hologic, Horizon A, APEX Software, Hologic Inc.). Scan sites modes and analysis are presented in Table S1. Animals were scanned under general anesthesia using the same densitometer on each occasion for animals in group 1 to group 5 prior to dosing and at weeks 12, 18, and 24. The initial scans acquired for each animal were compared with follow-up scans using the compare mode, when applicable. DXA-reported parameters included LS (L1-L4) area (cm^2^; data not shown), BMC (g), and BMD (g/cm^2^).

Temporal changes in DXA parameters were assessed for each treatment cycle. Percentage changes were calculated for the following.

- End of the first dosing cycle (week 12) relative to predose (week −1).
- End of the second dosing cycle (week 18) relative to the end of the first dosing cycle (week 12).
- End of the third dosing cycle (week 24) relative to the end of the second dosing cycle (week 18).
- End of the third cycle of dosing relative to predose to assess the cumulative effects of the 3 treatment cycles.

### Terminal procedures

Terminal procedures to collect tissue samples (including kidneys, L5, L2-L3, L4 vertebrae, and blood) and preparation of the tissues for assessment are described in detail in Supplementary Methods.

### Ex vivo micro-CT

Micro-CT was performed on the L5 vertebra individually collected from study animals in all treatment groups euthanized at week 24 (end of cycle 3) using a Scanco Medical AG micro-CT 100 (Scanco Medical) and analyzed using the 3D morphometry evaluation program.

L5 vertebrae were trimmed to obtain a standardized vertebral body positioned with the cranial side (identified with ink) facing the top of the holder and the dorsal side facing the front of the holder. The holder was filled with saline and covered with parafilm. The top reference line was set at the top of the bone (cranial aspect), and the bottom line was placed at the bottom of the bone (caudal aspect). The volume of interest (VOI) represents 104 consecutive complete slices and is delimited using automatic contouring. The evaluation method used is described below, using a 3D constrained Gaussian filter with finite filter support (2 voxels) and filter width (σ = 0.8). The images were then binarized to separate the object from the background using a global thresholding procedure. Scan and evaluation parameters are provided in Tables S2 and S3.

For each vertebra, one VOI was defined, contoured, and analyzed. Parameters evaluated using 3D morphometry are provided in Table S4.

### Histomorphometric assessments

Histomorphometric parameters listed in Table S5 were quantified/calculated on 7-μm-thick unstained sections of L2-L3 samples at week 18 (end of cycle 2), week 20 (2 wk of Scl-Ab treatment in cycle 3), and week 24 (6 wk of Scl-Ab treatment, end of cycle 3) by fluorescence microscopy. Remodeling-based bone formation (RBBF) and MBBF were assessed on 14-μm-thick unstained sections of L2-L3 samples at week 20 and week 24. The Cn osteoclastic surface (Cn OcS/BS) and Cn osteoblastic surface (Cn ObS/BS) were estimated on cathepsin K-immunostained sections of L4 from the same timepoints. Cn OcS/BS was defined as surfaces covered with cathepsin K-immunopositive cytoplasm. Cn ObS/BS was defined as surfaces that captured the earlier phases of a formative surface. These included surfaces covered with plump cuboidal osteoblasts with abundant cytoplasm and oval nuclei perpendicular to the bone surface and surfaces covered with flattened osteoblasts with clearly visible cytoplasm and oval nuclei typically parallel to the surface.

Surface-based measurements were performed by manual intercept counting using a linear ocular test system that was randomly rotated between fields to sample the bone surface and designated labeled surfaces. To assess MBBF and RBBF on Cn and Ec surfaces, sampled fields were viewed in fluorescence and brightfield polarized light to identify and characterize the labeled surface that included both single and double labels based on the morphologic characteristics of the cement line as modeling (smooth) or remodeling (scalloped) and expressed as percentage labeled surface and percentage bone surface as previously reported^12^^,^^18^ (Figure S1). Modeling/remodeling surfaces were quantified at 200×. Cn and Ec MS/BS were quantified at 100×.

### Statistics

All statistical tests were conducted at the 5% significance level. All pairwise comparisons were conducted using two-sided tests and reported at the 5% level, unless otherwise noted. Statistical analyses were conducted independently within each of cycles 1-3. Levene’s test was used to assess the homogeneity of group variances. The overall group effect was assessed via a one-way analysis of variance (ANOVA) F test if Levene’s test was not significant or via the Kruskal–Wallis test if it was significant. If the overall F test or Kruskal–Wallis test was found to be significant, the pairwise comparisons listed below were conducted using a *t-*test and Wilcoxon rank sum test, respectively. Adjustments for multiplicity of tests were made based on the square root of the number of pairwise comparisons, using the Bonferroni correction.

- OPG-Fc/OPG-Fc/Scl-Ab vs Veh/Veh/Scl-Ab (group 2 vs group 1)
- OPG-Fc/ZOL/Scl-Ab vs Veh/Veh/Scl-Ab (group 3 vs group 1)
- Aln/OPG-Fc/Scl-Ab vs Veh/Veh/Scl-Ab (group 4 vs group 1)
- OPG-Fc/OPG-Fc_Veh/Scl-Ab vs Veh/Veh/Scl-Ab (group 5 vs group 1)
- OPG-Fc/OPG-Fc/Scl-Ab vs OPG-Fc/ZOL/Scl-Ab (group 2 vs group 3)
- OPG-Fc/OPG-Fc/Scl-Ab vs OPG-Fc/OPG-Fc_Veh/Scl-Ab (group 2 vs group 5)
- OPG-Fc/ZOL/Scl-Ab vs Aln/OPG-Fc/Scl-Ab (group 3 vs group 4)

## Results

### In vivo densitometry

#### Percentage change in vertebral BMC and BMD during each treatment cycle

**Figure 2:**
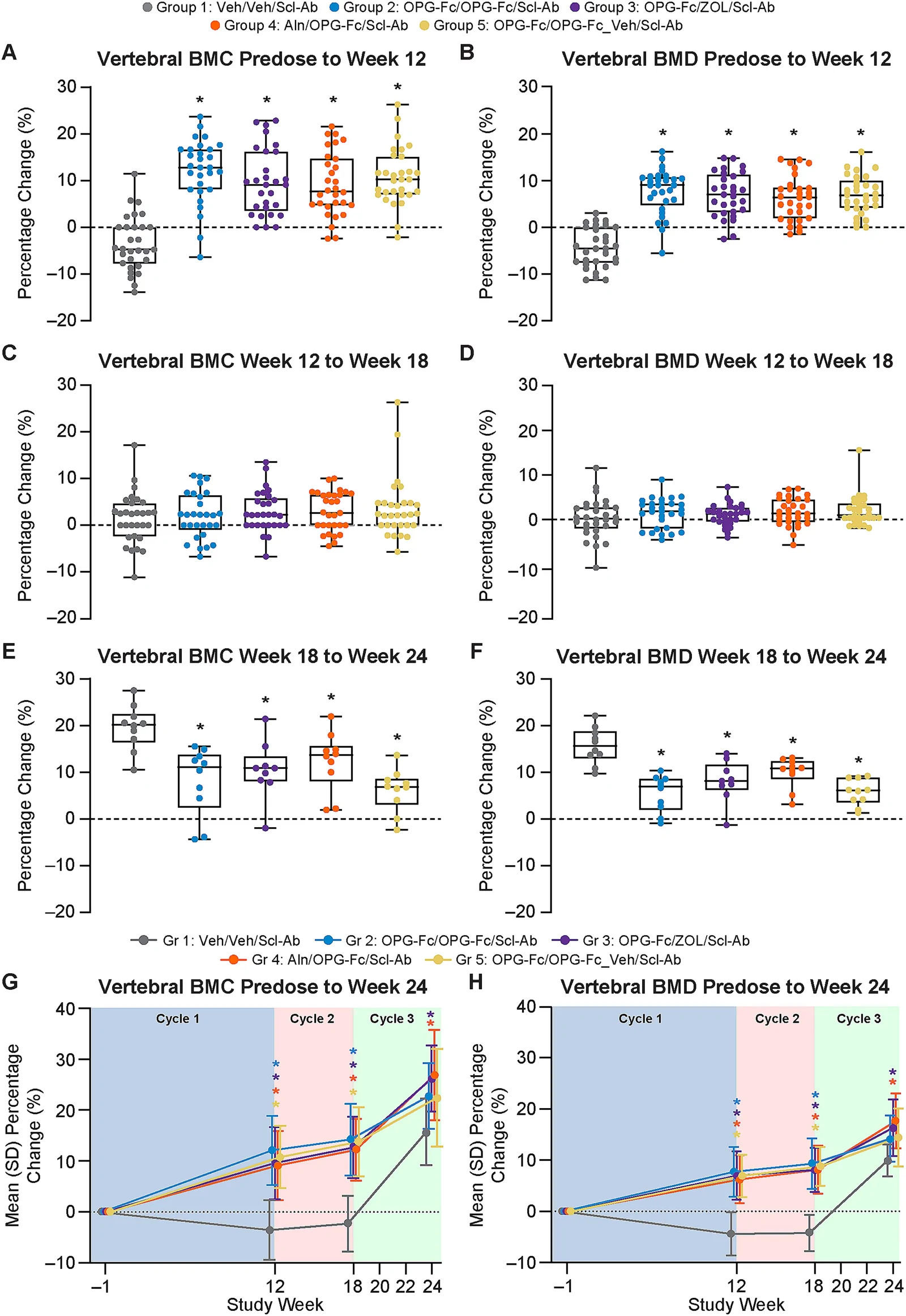
Percentage changes in (A, C, E, and G) vertebral BMC and (B, D, F, and H) vertebral BMD in ovariectomized rats administered various sequences of Veh, OPG-Fc, and a BP (ZOL or Aln) in cycles 1 and 2 before receiving Scl-Ab in cycle 3. Box plots show individual data with boxes indicating minimum and maximum, horizontal lines in the boxes indicating means, and vertical bars indicating SDs. ^*^p ≤ .05. Significantly different from group 1 (Veh/Veh/Scl-Ab). Aln, alendronate; BP, bisphosphonate; Gr, group; Scl-Ab, sclerostin antibody; Veh, vehicle; ZOL, zoledronic acid.

The first treatment cycle consisted of 1 group receiving Veh, 3 groups receiving OPG-Fc, and 1 group receiving Aln for 12 wk. The percentage change in vertebral BMC and BMD from predose to the end of cycle 1 was significantly greater in all groups administered antiresorptive treatment in cycle 1 compared with the group administered Veh (Figure 2A and B). The effects on BMC and BMD were not significantly different for predesignated between-group comparisons for groups receiving OPG-Fc or Aln in cycle 1.

The second treatment cycle lasted 6 wk and consisted of continuation of Veh (Veh/Veh) or OPG-Fc (OPG-Fc/OPG-FC), switched to ZOL (OPF-Fc/ZOL) or OPG-Fc (Aln/OPG-Fc), or continued on OPG-Fc for 2 wk followed by Veh for the last 4 wk of the cycle (OPG-Fc/OPG-Fc_Veh). The percentage change in vertebral BMC and BMD from the end of cycle 1 to the end of cycle 2 was generally minimal across all treatment groups (Figure 2C and D). Lack of further reduction in BMC and BMD in the Veh/Veh group suggests attenuation of the bone loss associated with ovariectomy in rats.^19^^,^^20^

During the third treatment cycle, all groups were switched to weekly dosing of Scl-Ab. The percentage change in vertebral BMC and BMD from the end of cycle 2 to the end of cycle 3 in response to Scl-Ab was significantly lower in all groups that received preceding cycles of various antiresorptive sequences compared with Veh/Veh/Scl-Ab indicating significant blunting of the early bone-building effects of Scl-Ab. There were no significant differences in the effects on BMC and BMD between the group comparisons that received prior antiresorptive sequences (Figure 2E and F).

The cumulative effects of various treatment sequences on vertebral BMC and BMD from predose to the end of cycle 3 are shown in Figure 2G, H. Only the groups that received a BP before or after OPG-Fc (OPG-Fc/ZOL/Scl-Ab or Aln/OPG-Fc/Scl-Ab) had a significantly greater cumulative percentage increase in BMC and BMD at the end of cycle 3 compared with Veh/Veh/Scl-Ab.

### Ex vivo micro-CT

#### Effect on vertebral cortical thickness and vertebral body bone volume at the end of cycle 3

**Figure 3:**
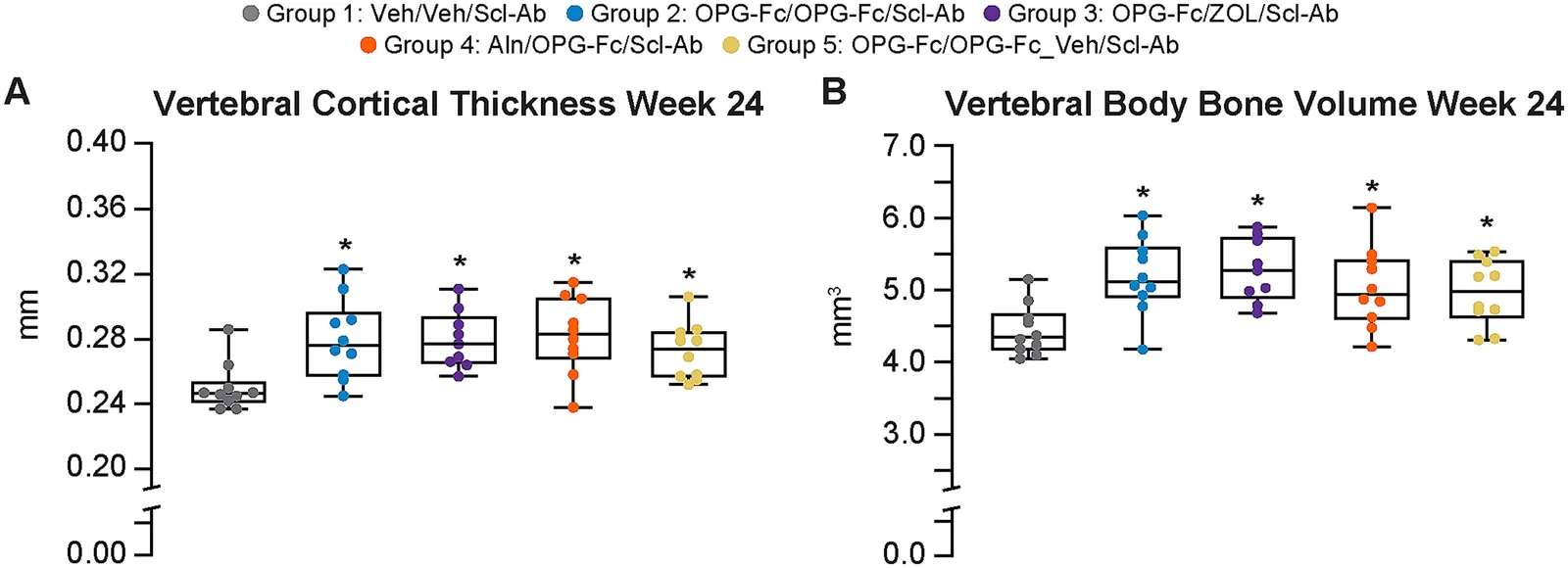
Effect on (A) vertebral cortical thickness and (B) vertebral body bone volume at the end of cycle 3 in ovariectomized rats administered various sequences of Veh, OPG-Fc, and a BP (ZOL or Aln) in cycles 1 and 2 before receiving Scl-Ab in cycle 3. Box plots show individual data with boxes indicating minimum and maximum, horizontal lines in the boxes indicating means, and vertical bars indicating SDs.^*^p ≤ .05. Significantly different from group 1 (Veh/Veh/Scl-Ab). Aln, alendronate; BP, bisphosphonate; Scl-Ab, sclerostin antibody; Veh, vehicle; ZOL, zoledronic acid.

At the end of cycle 3, an ex vivo analysis of the lumbar vertebrae revealed that cortical thickness was significantly greater in rats receiving prior antiresorptive sequences than in treatment-naïve rats receiving Scl-Ab (Figure 3A). Significantly higher vertebral body bone volume (Figure 3B) was observed in rats receiving prior antiresorptive treatment followed by Scl-Ab compared with rats in the Veh/Veh/Scl-Ab group, reflecting the effective inhibition of bone loss during cycle 1 and 2 due to ovariectomy in addition to the contribution, although blunted, of Scl-Ab in cycle 3. There were no significant differences in the effects on cortical thickness and vertebral body bone volume in the predesignated between-group comparisons that received prior antiresorptive sequences.

### Vertebral histomorphometry

#### Cn and Ec bone formation at end of cycle 2 (week 18)

**Figure 4:**
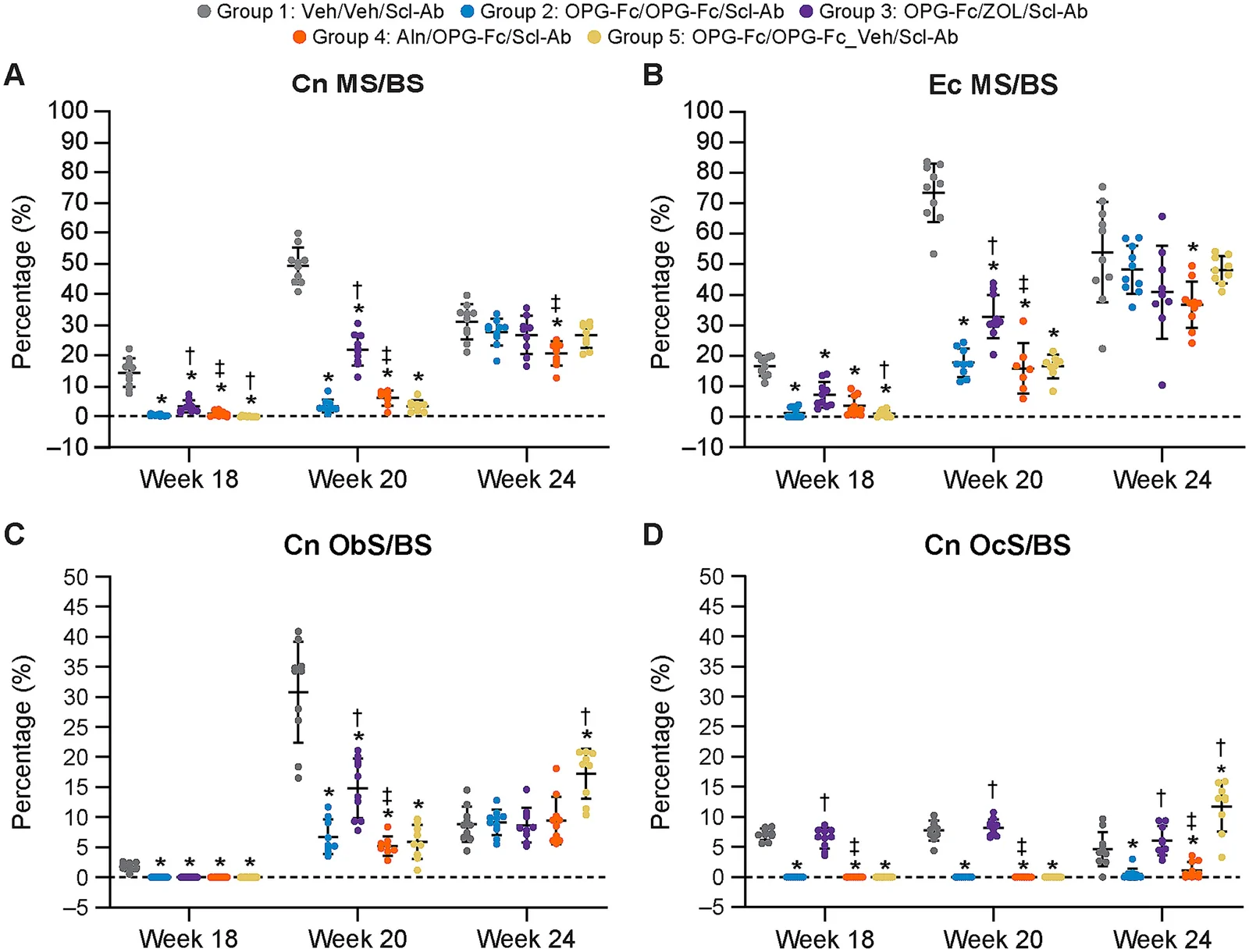
Effect on (A) Cn MS/BS, (B) Ec MS/BS, (C) Cn ObS/BS, and (D) Cn OcS/BS in ovariectomized rats administered various sequences of Veh, OPG-Fc, and a BP (ZOL or Aln) in cycles 1 and 2 before receiving Scl-Ab in cycle 3. Scatter plots show individual data with horizontal lines indicating means, and vertical bars indicating SDs. ^*^p ≤ .05. Significantly different from group 1 (Veh/Veh/Scl-Ab). †*p* ≤ .05: Group 3 (OPG-Fc/ZOL/Scl-Ab) or group 5 (OPG-Fc/OPG-Fc_Veh/Scl-Ab) significantly different from group 2 (OPG-Fc/OPG-Fc/Scl-Ab). ‡*p* ≤ .05. Group 4 (Aln/OPG-Fc/Scl-Ab) significantly different from group 3 (OPG-Fc/ZOL/Scl-Ab). Aln, alendronate; BP, bisphosphonate; BS, bone surface; Cn, cancellous; Ec, endocortical; MS, mineralizing surface; ObS, osteoblastic surface; OcS, osteoclastic surface; Scl-Ab, sclerostin antibody; Veh, vehicle; ZOL, zoledronic acid.

At the end of cycle 2, all groups receiving prior antiresorptive sequences had significantly lower Cn and Ec MS/BS compared with Veh/Veh/Scl-Ab, an anticipated consequence of the inhibition of bone resorption and bone turnover (Figure 4A and B). Although OPG-Fc/ZOL/Scl-Ab was significantly lower than Veh/Veh/Scl-Ab, this group had minimally higher values for Cn and Ec MS/BS compared with the other sequences that had received OPG-Fc in cycle 2, perhaps reflecting a minor resumption of turnover associated with the observed slight increase in CTx at the end of cycle 2 (BTM section in Supplementary Results; Figure S2). Cn ObS/BS generally paralleled Cn MS/BS (Figure 4C).

TRAcP5b in OPG-Fc/ZOL/Scl-Ab increased in cycle 2 compared to cycle 1 and was similar to Veh/Veh/Scl-Ab, indicating the recovery of osteoclastogenesis in cycle 2, evidence of OPG-Fc clearance (Figure 4D; Figure S2). In groups that received OPG-Fc in cycle 2, Cn OcS/BS was zero, consistent with the effects of a potent RANKL inhibitor on osteoclastogenesis.

#### Cn and Ec bone formation after 2 weeks of Scl-Ab in cycle 3 (week 20)

In Veh/Veh/Scl-Ab, after 2 wk of Scl-Ab treatment, there was a robust increase in Cn and Ec MS/BS (Figure 4A and B). In all groups receiving prior antiresorptive treatment sequences, Cn and Ec MS/BS were significantly lower compared with Veh/Veh/Scl-Ab, indicating that prior antiresorptive sequences blunted the early bone-forming response to Scl-Ab. Groups receiving antiresorptive treatments where OPG-Fc immediately preceded Scl-Ab (OPG-Fc/OPG-Fc/Scl-Ab and Aln/OPG-Fc/Scl-Ab) had more exaggerated blunting. Changes in Cn ObS/BS paralleled those in Cn MS/BS.

Cancellous OcS/BS was similar at week 18 across groups. Although Cn OcS/BS remained zero in groups that received OPG-Fc in cycle 2, in OPG-Fc/OPG-Fc_Veh/Scl-Ab, osteoclasts were present immediately under the vertebral endplates (not captured in OcS/BS) indicating that osteoclastogenesis was beginning to recover from OPG-Fc-mediated suppression in cycle 2 following a 4 wk treatment-free period.

**Figure 5:**
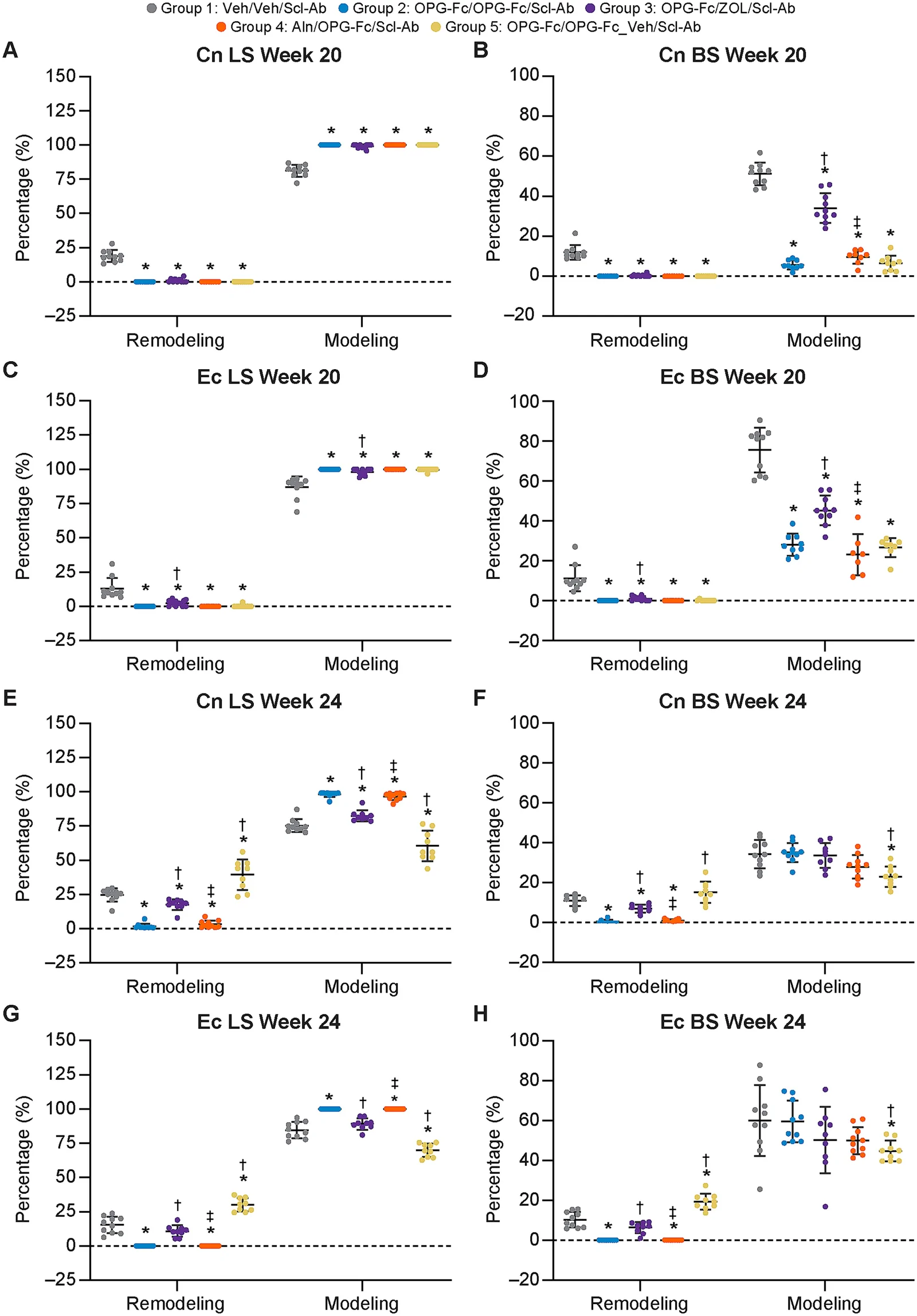
Effect on (A, B, E, and F) Cn and (C, D, G, and H) Ec bone histomorphometry in ovariectomized rats administered various sequences of Veh, OPG-Fc, and a BP (ZOL or Aln) in cycles 1 and 2 before receiving Scl-Ab in cycle 3. Scatter plots show individual data with horizontal lines indicating means, and vertical bars indicating SDs. ^*^p ≤ .05. Significantly different from group 1 (Veh/Veh/Scl-Ab). †*p* ≤ .05. Group 3 (OPG-Fc/ZOL/Scl-Ab) or group 5 (OPG-Fc/OPG-Fc_Veh/Scl-Ab) significantly different from group 2 (OPG-Fc/OPG-Fc/Scl-Ab). ‡*p* ≤ .05. Group 4 (Aln/OPG-Fc/Scl-Ab) significantly different from group 3 (OPG-Fc/ZOL/Scl-Ab). Aln, alendronate; BP, bisphosphonate; BS, bone surface; Cn, cancellous; Ec, endocortical; LS, labeled surface; MS, mineralizing surface; Scl-Ab, sclerostin antibody; Veh, vehicle; ZOL, zoledronic acid.

The percentages of RBBF and MBBF referent to labeled surface were similar on Cn and Ec surfaces in Veh/Veh/Scl-Ab. In groups that received preceding antiresorptive sequences, Cn MBBF/LS and Ec MBBF/LS constituted essentially all active bone formation (Figure 5A and C).

In all groups, when RBBF and MBBF was expressed referent to the total bone surface (Figure 5B and D), MBBF/BS predominated on Cn and Ec surfaces consistent with the early increase in bone formation in response to Scl-Ab although blunted by previous antiresorptive treatment. There was only modest blunting in OPG-Fc/ZOL/Scl-Ab (interposed ZOL) with greater suppression of MBBF/BS in groups transitioning directly from OPG-Fc to Scl-Ab. RBBF/BS was zero or <1% in groups with preceding antiresorptive treatments.

#### Cn and Ec bone histomorphometry with 6 weeks of Scl-Ab (end of cycle 3, week 24)

At the end of 6 wk of treatment with Scl-Ab, Cn and Ec MS/BS decreased in Veh/Veh/Scl-Ab relative to week 20, evidence of the anticipated attenuation of bone formation accompanied by a parallel decrease in Cn ObS/BS (Figure 4A-C). In the groups administered previous antiresorptive sequences, Cn and Ec MS/BS matched the Veh/Veh/Scl-Ab, except for Aln/OPG-Fc/Scl-Ab in which Cn and Ec MS/BS were significantly lower. Cancellous OcS/BS remained significantly lower in OPG-Fc/OPG-Fc/Scl-Ab and Aln/OPG-Fc/Scl-Ab compared with both Veh/Veh/Scl-Ab and OPG-Fc/ZOL/Scl-Ab, although values slightly increased compared with those at week 18, suggesting some recovery from OPG-Fc suppression of osteoclastogenesis. OPG-Fc/OPG-Fc_Veh/Scl-Ab showed the greatest increase in Cn OcS/BS with values exceeding both Veh/Veh/Scl-Ab and OPG-Fc/ZOL/Scl-Ab.

In Veh/Veh/Scl-Ab, following 6 wk of Scl-Ab, the percentages of RBBF/LS and MBBF/LS on Cn and Ec were similar to week 20 (Figure 5E and G). In OPG-Fc/ZOL/Scl-Ab, Cn and Ec MBBF/LS and RBBF/LS were generally similar to Veh/Veh/Scl-Ab with Cn and Ec RBBF/LS increasing from week 20. In OPG-Fc/OPG-Fc/Scl-Ab and Aln/OPG-Fc/Scl-Ab, MBBF/LS remained the major contributor to labeled surface. In contrast in OPG-Fc/OPG-Fc_Veh/Scl-Ab, RBBF/LS now constituted approximately 30%-40% of active labeled surfaces (Figure 5E and G).

When expressed referent to total bone surfaces, Cn and Ec RBBF/BS displayed the same pattern of group differences as observed referent to labeled surface (Figure 5F and H). In Veh/Veh/Scl-Ab, Cn and Ec RBBF/BS were similar to week 20 with lower Cn MBBF/BS and Ec MBBF/BS, consistent with attenuation of bone formation. Cn and Ec MBBF/BS were not significantly different among the groups except in the OPG-Fc/OPG-Fc_Veh/Scl-Ab group, where RBBF/BS now represented an increased percentage of bone-forming surfaces. The increase in RBBF/BS in this group aligns with the significant increases in Cn OcS/BS and progressive increase in resorption markers beginning at week 20 (Figure S3) reflecting the clearance of OPG-Fc early in cycle 3 and rebound resorption/remodeling.

Qualitatively, the morphologic features of active MBBF sites differed between treatment groups at week 20. In Veh/Veh/Scl-Ab and OPG-Fc/ZOL/Scl-Ab groups, the active bone-forming sites were characterized by fluorochrome labels that stretched along considerable lengths of Cn and Ec surfaces (Figure S3A and B). In contrast, in groups that received OPG-Fc immediately preceding Scl-Ab, most MBBF sites were characterized by small club-shaped protrusions of actively forming bone (Figure S3C). The group differences in the morphology of active modeling formation sites noted at week 20 persisted at week 24. In groups where OPG-Fc immediately preceded Scl-Ab, the increase in MBBF from week 20 was manifested by the increased frequency of mini-modeling sites (Figure S3D). In the OPG-Fc/OPG-Fc Veh /Scl-Ab group, mini-modeling predominated at week 20 but by week 24, a significant percentage of the labeled bone surface was RBBF; the morphology of remodeling formation surfaces was not affected by prior OPG-Fc treatment.

## Discussion

This study was designed to investigate the effects of preceding sequences of BPs and OPG-Fc on early bone formation response to Scl-Ab in vertebrae from mature ovariectomized rats by assessing changes in vertebral BMC and BMD, vertebral dynamic histomorphometry, and serum BTMs. The specific treatment sequences were designed to model sequences of antiresorptive treatment and romosozumab that have been used in clinical settings for treatment of postmenopausal women with osteoporosis and are at high risk for fracture.

As expected during cycles 1 and 2, all antiresorptive sequences were equally effective in preserving and increasing spinal BMC and BMD compared with Veh/Veh/Scl-Ab, effectively blocking the progressive loss of bone due to ovariectomy and aging and reducing remodeling space. All antiresorptive sequences reduced TRAcP 5b and CTx. TRAcP 5b was markedly reduced in cycles when OPG-Fc was administered. Less suppression occurred with Aln in cycle 1 and TRAcP 5b was restored to Veh levels when transitioned to ZOL from OPG-Fc, indicating resumption of osteoclastogenesis. At the end of cycle 3, the increase in BMC and BMD in response to Scl-Ab was significantly blunted in all groups receiving prior antiresorptive treatments.

In cycle 3, the response to Scl-Ab treatment in the Veh/Veh/Scl-Ab group displayed the expected temporal pattern of bone formation over the 6-wk period. There was an early robust increase in MBBF at week 20 with greater effects on Ec than on Cn, consistent with findings in humans and nonhuman primates.^10^^,^^12^^,^^18^ Bone formation attenuated at week 24, consistent with the self-regulation of bone formation that occurs with Scl-Ab.^21^ The increased bone formation was reflected in a correlative increase in P1NP. RBBF was unaffected across the treatment period on both Cn and Ec surfaces, consistent with the limited effects of Scl-Ab on bone resorption in rats and monkeys at clinically relevant exposures and in contrast to the sustained moderate suppression of bone resorption in humans.^2^^,^^10^^,^^21–24^ As a consequence of the increase in MBBF, vertebral BMC and BMD showed expected increases.

In all groups with preceding antiresorptive sequences, the early bone-forming response to Scl-Ab in cycle 3 (increased MBBF) at week 20 was significantly blunted on Cn and Ec surfaces, with the least blunting occurring when ZOL was interposed between OPG-Fc and Scl-Ab. In this group, there was a significant increase in bone formation (MBBF) compared with week 18, but the increase was significantly less than in Veh/Veh/Scl-Ab. Values for MBBF were generally sustained at week 24 ultimately matching values with those in Veh/Veh/Scl-Ab. The contribution of RBBF was significantly less in OPG-Fc/ZOL/Scl-Ab compared with Veh/Veh/Scl-Ab at week 20, indicating continued suppression of bone resorption by the preceding cycle of ZOL with partial or complete restoration of RBBF to Veh/Veh/Scl-Ab levels at week 24.

At week 20, groups that had received OPG-Fc immediately preceding Scl-Ab had the greatest suppression of tissue-based bone formation parameters on Cn and Ec surfaces (MS/BS, ObS/BS, and MBBF) in response to Scl-Ab. RBBF was essentially zero, reflecting the profound suppression of resorption and bone remodeling by OPG-Fc.

By week 24, in groups that received OPG-Fc immediately preceding Scl-Ab, the suppressive effects of preceding OPG-Fc treatment on the bone-forming effects of Scl-Ab appeared to abate. In these 3 groups, Ec and Cn MS/BS increased from week 20 with values approaching, or similar to, Veh/Veh/Scl-Ab and OPG-Fc/ZOL/Scl-Ab with the increase in bone formation almost exclusively due to increased MBBF except in OPG-Fc/OPG-Fc_Veh/Scl-Ab. In the OPG-Fc/OPG-Fc_Veh/Scl-Ab group, the increase was due to an increase in both RBBF and MBBF. The increase in remodeling is consistent with the increased Cn OcS/BS and changes in BTMs across cycle 3 with TRAcP 5b and CTx increasing progressively from week 20, reflecting earlier clearance of OPG-Fc as a consequence of the preceding treatment-free period. This pattern of rebound in resorption markers has been reported with denosumab discontinuation in humans.^25–27^ Scl-Ab did not effectively blunt this rebound resorption due to the limited effects of Scl-Ab on resorption at clinically relevant exposures in rats as previously noted.^19–22^ The progressive increase in P1NP and CTx from week 18 to week 24 in OPG-Fc/OPG-Fc_Veh/Scl-Ab is similar to the progressive increases observed in subjects transitioned from denosumab to romosozumab in the phase 2 extension trials suggesting potential competing effects of MBBF and RBBF on bone mass accrual potentially contributing to the more limited increase in spinal BMD in the clinical trial.^5^^,^^6^

In OPG-Fc/OPG-Fc/Scl-Ab, the increase in bone formation (MBBF) was associated with an increase in TRAcP 5b and CTx at week 24 compared with week 22, suggesting some level of clearance of OPG-Fc and resumption of osteoclastogenesis supported by the small nonsignificant increases in Cn OcS/BS. Rebound resorption would be expected to occur with time in this group similar to OPG-Fc/OPG-Fc_Veh/Scl-Ab. In Aln/OPG-Fc/Scl-Ab, TRAcP 5b and CTx remained suppressed at week 24. The greater suppression of resorption markers at week 24 in Aln/OPG-Fc/Scl-Ab compared with OPG-Fc/ZOL/Scl-Ab may be consequence of the longer duration of BP exposure in Aln/OPG-Fc/Scl-Ab that received 12 wk of Aln in cycle 1 prior to OPG-Fc. Both BP regimens were effective in blunting the effects of OPG-Fc clearance on resorption during cycle 3 but additional studies with a longer duration cycle 3 would be required to determine if extended BP treatment prior to a RANKL inhibitor offers more protection from rebound resorption over time. Although the doses of ALN and ZOL were based on published rodent studies to model effects reported in humans, this question is best addressed clinically with approved doses and dosing regimens of these BPs.

Differences in the morphologic features of the actively forming modeling-based bone surfaces in response to Scl-Ab were noted among the treatment groups. In groups where OPG-Fc immediately preceded Scl-Ab, sites of actively forming bone were consistent with mini-modeling at weeks 20 and 24. Mini-modeling is a microscopic focal area of bone formation without previous resorption.^28^^,^^29^ In response to high doses of vitamin D analogs in the rats^30^ and growing rats,^31^ exaggerated mini-modeling formation will result in these button-like protrusions of MBBF. A treatment-free period in the OPG-Fc/OPG-Fc_Veh/Scl-Ab group, where OPG-Fc was clearing early in cycle 3 did not alter the tissue-level response qualitatively at week 20 but by week 24 there was a significant increase in RBBF, where RBBF formation surfaces were morphologically similar to the Veh/Veh/Scl-Ab and OPG-Fc/ZOL/Scl-Ab groups at both 20 and 24 wk.

The basis for the differential effects of preceding antiresorptive treatment on the initial formation response to Scl-Ab is unclear. One possible difference is that some level of bone turnover needs to be present to mediate the early effects of Scl-Ab on the osteoblast lineage, specifically maintenance of a responsive osteoprogenitor pool to sustain MBBF subsequent to initial activation of lining cells.^32–35^ Although the bone formation response to Scl-Ab with preceding ZOL was blunted compared with treatment-naïve rats, it was significantly greater than in groups with OPG-Fc immediately preceding Scl-Ab, where bone turnover was totally suppressed. The progenitor pool would be expected to be limited in the absence of bone turnover as studies in mice have shown that the quantity of committed osteoprogenitors correlates with the rate of bone turnover. A recent detailed analysis of osteoprogenitor populations defined by single cell RNA sequencing reported that OPG-Fc treatment in mice reduced a specific subpopulation of peritrabecular preosteoblasts suggesting, to some extent, preosteoblasts are dependent on bone resorption and associated coupling factors to sustain their populations.^36^ Investigations into the effects on the osteoprogenitor pool would be an avenue for future studies.

Another possible difference is the effect of preceding antiresorptive treatments on the osteoclast population. Although the osteocyte is the primary cellular mediator of the effects of activation of Wnt signaling by inhibition of sclerostin,^24^^,^^37^ the role of the osteoclast in the bone-forming effects of Scl-Ab remains unclear. Literature supports that osteoclasts do more than resorb bone. Mouse models with mutations that result in an absence of osteoclasts are associated with impaired bone formation in contrast with models where bone resorption is impaired but osteoclast numbers are maintained.^38–41^ Osteoclast-derived factors play an important role in coupling resorption to bone formation in the remodeling unit such as osteoclast-derived leukemia inhibitory factor that is reported to suppress sclerostin.^42^ In OPG-Fc/ZOL/Scl-Ab, Cn OcS/BS was similar to the Veh/Veh/Scl-Ab group but osteoclasts were functionally impaired based on CTx. In groups transitioning directly from OPG-Fc that had the greatest blunting of the bone formation response, Cn OcS/BS was zero. Hence, a role of osteoclast signaling in the early bone-forming response to Scl-Ab cannot be excluded. In contrast, studies in mice have demonstrated that lack of osteoclasts and suppression of bone remodeling are not required for an anabolic response to suprapharmacologic doses of Scl-Ab.^43^ The basis for the different responses in rats versus mice is unclear.

Some of the blunting of the bone-forming effects of Scl-Ab following a BP may be related to the effects of BPs on the osteoblast lineage. BPs have been reported to prolong the reversal phase of the remodeling cycle and impair the onset of bone formation at the remodeling site and prolong the terminal phases of the formative site in the remodeling unit.^38^^,^^44–46^ The attenuated bone-forming response to Scl-Ab when immediately preceded by a BP may, in part, be a consequence of effects on osteoblast recruitment. In addition, although osteoclasts are present, they are functionally impaired in terms of bone resorption capabilities which may impact in some way their signaling functions.

### Limitations

A limitation of our study was the duration of treatment in cycle 3. Six weeks captures the most robust effect of Scl-Ab in bone formation with evidence of expected attenuation, but the duration of treatment in cycle 3 did not capture the complete effect of Scl-Ab on bone formation, predicted to require approximately 12 wk at this dose/exposure^24^ or potential effects of certain sequences on subsequent rebound resorption. The duration was sufficient to differentiate the effects of preceding treatments on the early formation response to Scl-Ab. Additionally, OPG-Fc was used as a surrogate for denosumab, the latter shown to be more potent and have a longer duration of effect.^47^ An additional limitation of the study is that the rat model precludes analyses of the effects of the treatment sequences on intracortical remodeling. Because long bone was not evaluated, it is not known whether the treatment sequences elicited a different response in this compartment. The vertebrae was chosen, because it has abundant cancellous bone surfaces compared with cancellous surfaces in long bone that facilitates surface-based measurements and avoids issues of a physeal plate.

### Conclusion

Results from our study in ovariectomized rats treated with sequences of BPs and OPG-Fc that preceded treatment with Scl-Ab revealed that the initial bone-forming response to Scl-Ab at the tissue level is impaired by prior antiresorptive sequences but are least suppressed when Scl-Ab is preceded by ZOL. The suppression of the early MBBF was significantly greater when OPG-Fc immediately preceded Scl-Ab. Data in this study recapitulate aspects of previous clinical trials. In STRUCTURE, a minimum of 3 yr of Aln pretreatment was associated with attenuated bone mass accrual,^4^ similar to responses observed with OPG-Fc/ZOL/Scl-Ab group. In contrast to groups transitioning directly from OPG-Fc to Scl-Ab, the interposed cycle of ZOL restored at least a moderate bone-forming response. This may suggest that in postmenopausal patients transitioning from denosumab to romosozumab, an interposed cycle of BP may improve the tissue-level response to Scl-Ab. In the phase 2 extension studies in subjects transitioned from denosumab to romosozumab, the early increase in P1NP was blunted with P1NP and CTx progressively increasing over a 12-mo period when transitioned to romosozumab. This is in contrast to the early robust peak in P1NP followed by attenuation observed in romosozumab antibody-naïve subjects across RCTs.^5^ An early increase in P1NP in subjects transitioning from denosumab to romosozumab, although generally attenuated compared with treatment-naïve subjects, likely reflects activation of MBBF. However, the continued increase in P1NP accompanied by increased CTx would support increased bone remodeling being a significant contributor to the progressive increase in P1NP as a consequence of clearance of denosumab. Positive effects of romosozumab on bone formation in early Ec remodeling formative sites would also contribute to P1NP levels.^11^ These changes resemble those observed in OPG-Fc/OPG-Fc_Veh/Scl-Ab in cycle 3. Increased MBBF and increased RBBF would have competing effects on bone mass accrual. Although romosozumab is a modest inhibitor of resorption, BP treatment prior to or following denosumab before transitioning to romosozumab, may offer more protection from the increase in resorption and remodeling associated with denosumab clearance. However, the most robust bone-building effect in response to Scl-Ab was observed in treatment-naïve rats.

## Supplementary Material

Supplementary_Material_ziag115

## Data Availability

Qualified researchers may request data from Amgen clinical studies. Complete details are available at the following: http://www.amgen.com/datasharing.
