# Supplementary material for: Early bone-formation response to sclerostin antibody is altered by prior antiresorptive treatment in ovariectomized rats
Source: JBMR Plus. 2026 Jul 21;10(9):ziag115. doi: 10.1093/jbmrpl/ziag115 (PMC13496399; doi:10.1093/jbmrpl/ziag115)
Supplement: Supplementary file 1 [file supplementary_material_ziag115.pdf]

SUPPLEMENTARY MATERIAL

Early Bone-Formation Response to Sclerostin Antibody is Altered by Prior Antiresorptive Treatment in Ovariectomized Rats

Agathe Bédard,[1] Denise Dwyer,[2*] Melanie Felx,[1] Martin Guillot,[1] Amy Bourdeau,[3] Marina Stolina,[2] Jen Timoshanko,[4] Zhigang Yu,[5] Mary Oates,[5] Rogely Waite Boyce,[3] Serge Ferrari[6]

[1]Charles River Laboratories Montreal ULC, 22022 Trans-Canada Hwy, Senneville, Quebec H9X 3R3, Canada; [2]Cardiometabolic Disorders, Amgen Inc., One Amgen Center Drive, Thousand Oaks, CA 91320, USA; [3]Translational Safety and Bioanalytical Sciences, Amgen Inc., One Amgen Center Drive, Thousand Oaks, CA 91320, USA; [4]UCB, 208 Bath Rd, Slough SL1 3WE, UK; [5]Global Development, Amgen Inc., One Amgen Center Drive, Thousand Oaks, CA 91320, USA; [6]Geneva University Hospital, Rue Gabrielle-Perret-Gentil 4, 1205 Geneva, Switzerland

***Corresponding Author**

Denise Dwyer; Cardiometabolic Disorders, Amgen Inc., One Amgen Center Drive, Thousand Oaks, CA 91320, USA. Email: ddwyer@amgen.com

Supplementary Methods

Bone Turnover Marker (BTM) Assessments

Enzyme immune assays were used to measure serum concentrations of the bone resorption markers TRAcP 5b (RatTRAP Assay) and CTx (RatLaps EIA) and bone formation marker P1NP (Rat/Mouse P1NP EIA). All kits were manufactured by Immunodiagnostic Systems Inc (Gaithersburg, MD, USA) and used according to the manufacturer's instructions.

Temporal changes in these BTMs were assessed for each treatment cycle and displayed as absolute concentrations and percentage changes between treatment cycles. Percentage changes were calculated for the intervals as follows using a general equation of (postdose – predose)/predose*100:

- End of the first dosing cycle (Week 12) relative to predose (week –1)
- End of the second dosing cycle (Week 18) relative to the end of the first dosing cycle (Week 12)
- Third dosing cycle timepoints (Weeks 20, 22, and 24) relative to the end of the second dosing cycle (Week 18)

Terminal Procedures and Preparation of Tissues for Assessment

Ten and three days prior to necropsy, rats received 8 mg/kg calcein green SC in 0.5% bicarbonate. Ten rats per group were euthanized by exsanguination under isoflurane anesthesia at Week 18, Week 20, and Week 24, and subjected to a partial necropsy examination consisting of examining the lumbar vertebra, kidneys, femur, and masses noted in life. Because of the advanced age of the rats, the kidneys were examined histologically to rule out the potential presence of underlying chronic kidney disease, a common finding in aged rats that could

confound bone histomorphometric assessments. Kidneys were processed routinely in paraffin, sectioned, and stained with hematoxylin & eosin and examined by a board-certified veterinary pathologist. Minimal age-related tubular changes were occasionally noted but there were no findings considered to be of sufficient severity to have significantly impaired kidney function or affect serum creatinine.

L5 was removed, cleaned of soft tissue, wrapped in saline soaked gauze, and frozen at –20°C for the micro-computed tomography (micro-CT) analysis. L2–L3 vertebrae were removed as a segment and L4 was removed separately, and both segments were cleaned of soft tissue, fixed in 10% neutral buffered formalin for 48 hours, and then transferred to 70% ethanol. Following dehydration in alcohol and clearing in xylene, the L2–L3 segment was infiltrated and embedded in methylmethacrylate. Unstained sections from three levels, approximately 200 µm apart, were prepared to evaluate the Cn bone in the vertebral body. Seven-micrometer-thick unstained sections were prepared for dynamic histomorphometry, and Goldner-stained sections were used for qualitative evaluation. Additional 14-µm-thick unstained sections were prepared from samples collected at Week 20 and Week 24 for assessment of MBBF and remodeling-based bone formation (RBBF). L4 collected at Weeks 18, 20, and 24 was decalcified in Immunocal™ (StatLab, McKinney, TX, USA), processed in paraffin, sectioned at 5 µm, and immunostained for cathepsin K (rabbit monoclonal anti-CTSK, Abcam, Waltham, MA, USA; ab300569). Staining was performed using overnight Diva Decloaker antigen retrieval (Biocare, Pacheco, CA, USA), a BSA blocking kit (Fisher, Waltham, MA, USA; Fisher number 37520), and

goat anti-rabbit HRP (Abcam, Waltham, MA, USA; ab214880). Slides were stained using a DAB kit (Abcam, Waltham, MA, USA; ab64238) and counterstained with hematoxylin.

Supplementary Results

Temporal Changes in Serum BTMs During Each Cycle of Treatment

By the end of the first 12-week treatment cycle reductions were seen in TRAcP 5b and CTx (**Supplementary Figure 2**) in all antiresorptive treatment groups compared with the Veh-treated group; reduction in TRAcP 5 was greater with OPG-Fc. There was no meaningful effect of any antiresorptive treatment during treatment cycle 1 on P1NP compared with Veh-treated.

In cycle 2 suppression of resorption markers was sustained with greater suppression with OPG-Fc. Notable was the rebound in serum TRAcP 5b levels when transitioning from OPG-Fc to ZOL in cycle 2. At the end of cycle 2, bone formation marker P1NP was reduced in almost all antiresorptive treatment groups compared with the Veh-treated group.

In cycle 3, when all groups received Scl-Ab, all groups experienced increases in P1NP at week 20. The P1NP increase observed at week 20 showed some attenuation beginning at week 22 in Veh/Veh/Scl-Ab and OPG-FC/Zol/Scl-Ab consistent with the known attenuation of bone formation with Scl-Ab. At week 22, the increase was sustained in OPG-FC/OPG-Fc/Scl-Ab and increased in Aln/OPG-Fc/Scl-Ab and OPG-Fc/OPG-Fc_Veh/Scl-Ab, By week 24, only OPG-Fc/OPG-Fc_Veh/Scl-Ab which experienced a 4-week drug holiday after OPG-Fc treatment and prior to the Scl-Ab treatment , had a greater increase in P1NP levels compared with the Veh/Veh/Scl-Ab group.

CTx was significantly decreased across cycle 3 in Aln/OPG-Fc/Scl-Ab compared with Veh/Veh/Scl-Ab reflecting continued suppression of resorption following the 12-week Aln treatment during cycle 1. OPG-Fc/OPG-Fc_Veh/Scl-Ab had significant increases in CTx across cycle 3 compared with Veh/Veh/Scl-Ab. correspond to the clearance of OPG-Fc. No meaningful changes in CTx levels were noted in OPG-Fc/ZOL/Scl-Ab and was similar to Veh/Veh/Scl-Ab..

In cycle 3, TRAPcP5b remained suppressed at weeks 20 and 22 in OPG-Fc/OPG-Fc/Scl-Ab Aln/OPG-Fc/Scl-Ab.At week 24, minor increases in TRAcP 5b were noted in these two groups compared with week 22. In OPG-Fc/ZOL/Scl-Ab, TRAcP 5b was generally similar to Veh/Veh/Scl-Ab across cycle 3. The most striking effect on TRAcP 5b during cycle 3 was seen in OPG-Fc/OPG-Fc_Veh/Scl-Ab, with a robust rebound from weeks 18 to 24. Collectively, the increases in serum P1NP, CTx, and TRAcP 5b in OPG-Fc/OPG-Fc_Veh/Scl-Ab during cycle 3 are reflective of increased bone remodeling related to clearance of OPG-Fc.

Supplementary Table 1. DXA scan sites, scan modes, and analysis

Scan Site	Scan Type	Scan Mode	Analysis Mode
Lumbar spine	Small animal regional hi-res	High definition	Subregion hi-res

DXA, dual energy x-ray absorptiometry; hi-res, high resolution.

Supplementary Table 2. Scan parameters

		**L5 Vertebral Body**
General	Holder type	U50822, 9 mm x 78 mm
	Evaluation	Default
X-ray settings	Energy/intensity	70 kVp, 114 µA, 8 W
	Filter	AL 0.5 mm
	Calibration	70 kVp, 0.5 mm A1, BH: 120 mg HA/cc
CT scans	Resolutions	Medium
	FOV/diameter (mm)	10.0
	Voxel size (µm)	10.0
	Number of slices	530
	Integration time (ms)	200
	Average data	1

CT, computed tomography; FOV, field of view; ms, millisecond.

Supplementary Table 3. Evaluation analysis parameters

	**Evaluation**
Sigma	0.8
Support	2
Lower threshold	260
Upper threshold	1000

Supplementary Table 4. Micro-CT reported parameters

**Results**	**Abbreviation/Formula**	**Unit**
L5 cortical region		
Total volume	TV	mm^3
Bone volume	BV	mm^3
Relative bone volume	BV/TV	%
Cortical region vBMD	vBMD (mean 1)	Mg HA/cm^3
Cortical tissue mineral density	TMD (mean 2)	Mg HA/cm^3
Cortical region BMC	TV*Mean 1/1000	Mg HA
Cortical thickness	Ct.th	Mm

BMC, bone mineral content; CT, computed tomography; vBMD, volumetric bone mineral density.

Supplementary Table 5. Parameters quantified by fluorescent microscopy

**Parameter (%)**	**Cancellous**	**Endocortical**
Single labeled surface	sLS/BS	sLS/BS
Double labeled surface	dLS/BS	dLS/BS
Mineralizing surface	MS/BS	MS/BS
Modeling-based bone formation BS referent	MBBF/BS	MBBF/BS
Remodeling-based bone formation BS referent	RBBF/BS	RBBF/BS
Modeling-based bone formation labeled surface referent	MBBF/LS	MBBF/LS
Remodeling-based bone formation labeled surface referent	RBBF/LS	RBBF/LS

Supplementary Figure 1. Examples of modeling- and remodeling-based bone formation

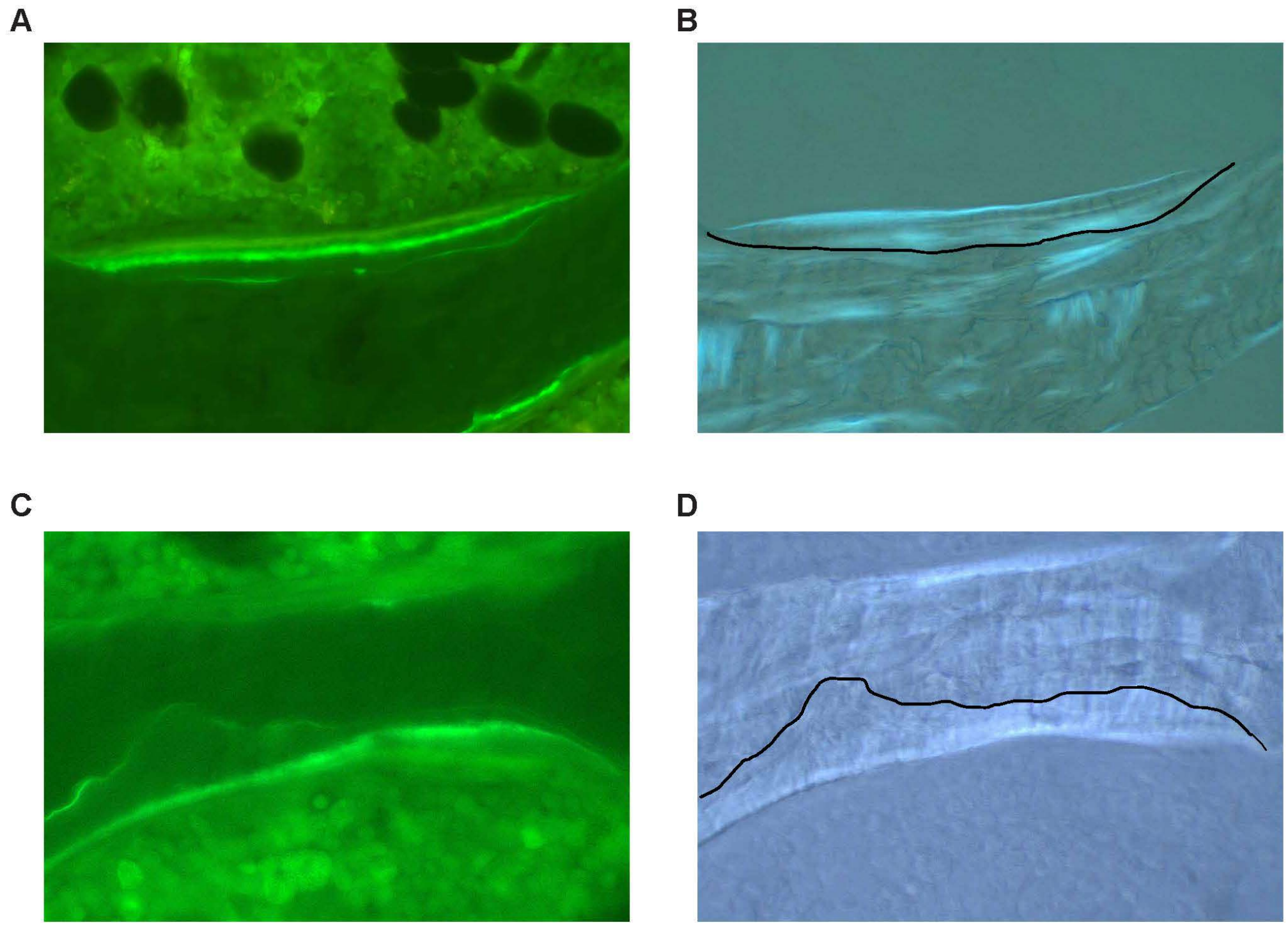

Example of a modeling-based formation surface from a rat in group 1 (Veh/Veh/Scl-Ab) at week 20 viewed in (A) fluoresence and (B) polarized brightfield. Example of a remodeling-based formation site from a rat in group 5 (OPG-Fc/OPG-Fc_Veh/Scl-Ab) at week 24 viewed in (C) fluoresence and (D) polarized brightfield. Original magnification 200x.
Scl-Ab, sclerostin antibody; Veh, vehicle.

Supplementary Figure 2. Absolute and percentage changes in serum BTMs

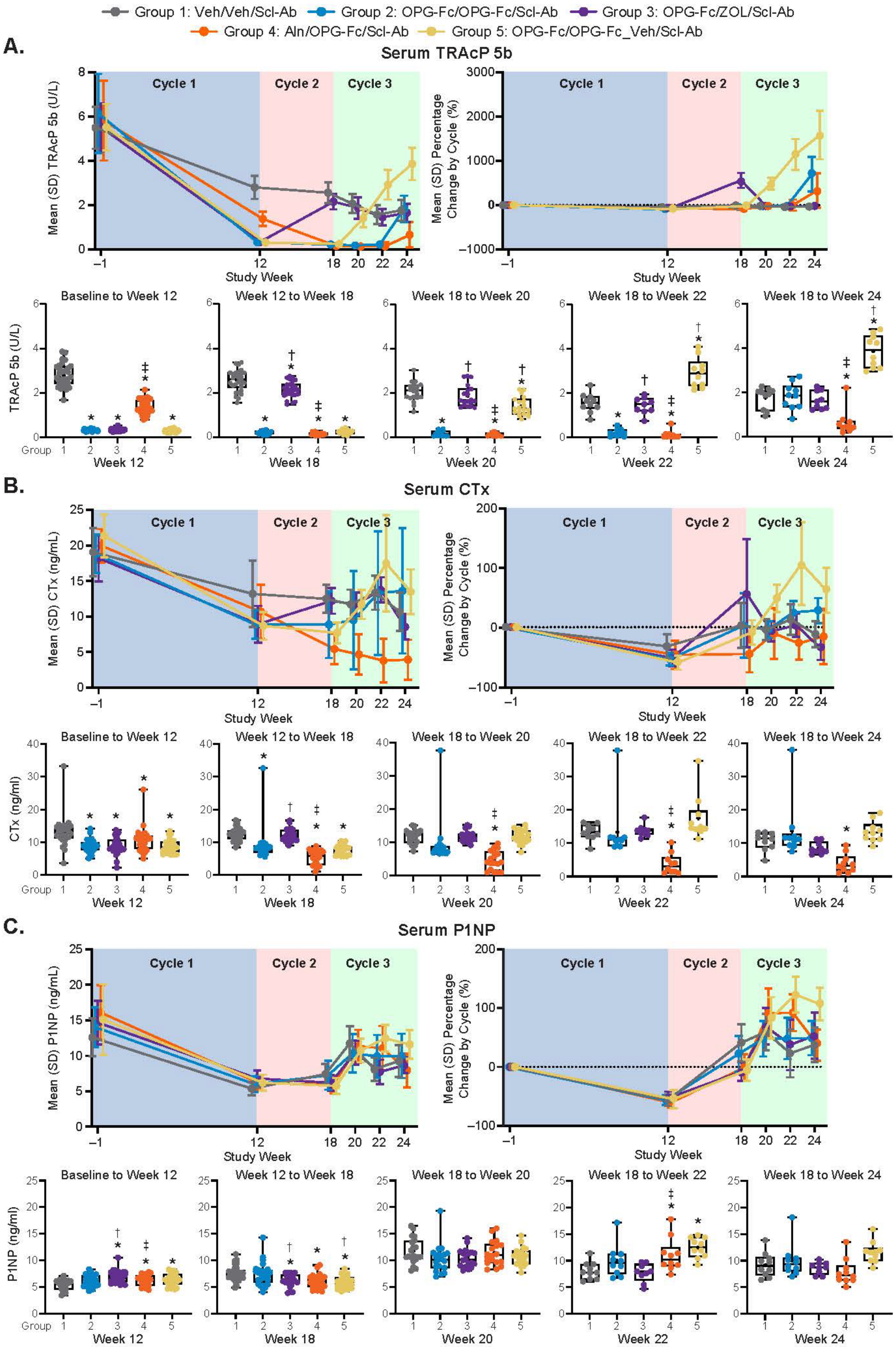

(A) TRAcP 5b, (B) CTx, and (C) P1NP in ovariectomized rats administered various sequences of Veh, OPG-Fc, and a BP (ZOL or Aln) in cycles 1 and 2 before receiving Scl-Ab in cycle 3. Box plots

show individual data with boxes indicating minimum and maximum, horizontal lines in the boxes indicating means, and vertical bars indicating SDs.
*$P \leq 0.05$: Significantly different from group 1 (Veh/Veh/Scl-Ab); †$P \leq 0.05$: Group 3 (OPG-Fc/ZOL/Scl-Ab) or group 5 (OPG-Fc/OPG-Fc_Veh/Scl-Ab) significantly different from group 2 (OPG-Fc/OPG-Fc/Scl-Ab); ‡$P \leq 0.05$: Group 4 (Aln/OPG-Fc/Scl-Ab) significantly different from group 3 (OPG-Fc/ZOL/Scl-Ab).
Aln, alendronate; BP, bisphosphonate; BTM, bone turnover marker; CTx, C-terminal telopeptide of type I collagen; P1NP, N-terminal propeptide of type I collagen; Scl-Ab, sclerostin antibody; SD, standard deviation; TRAcP 5b, tartrate-resistant acid phosphatase form 5b; Veh, vehicle; ZOL, zoledronic acid.

Supplementary Figure 3. Morphologic features of MBBF.

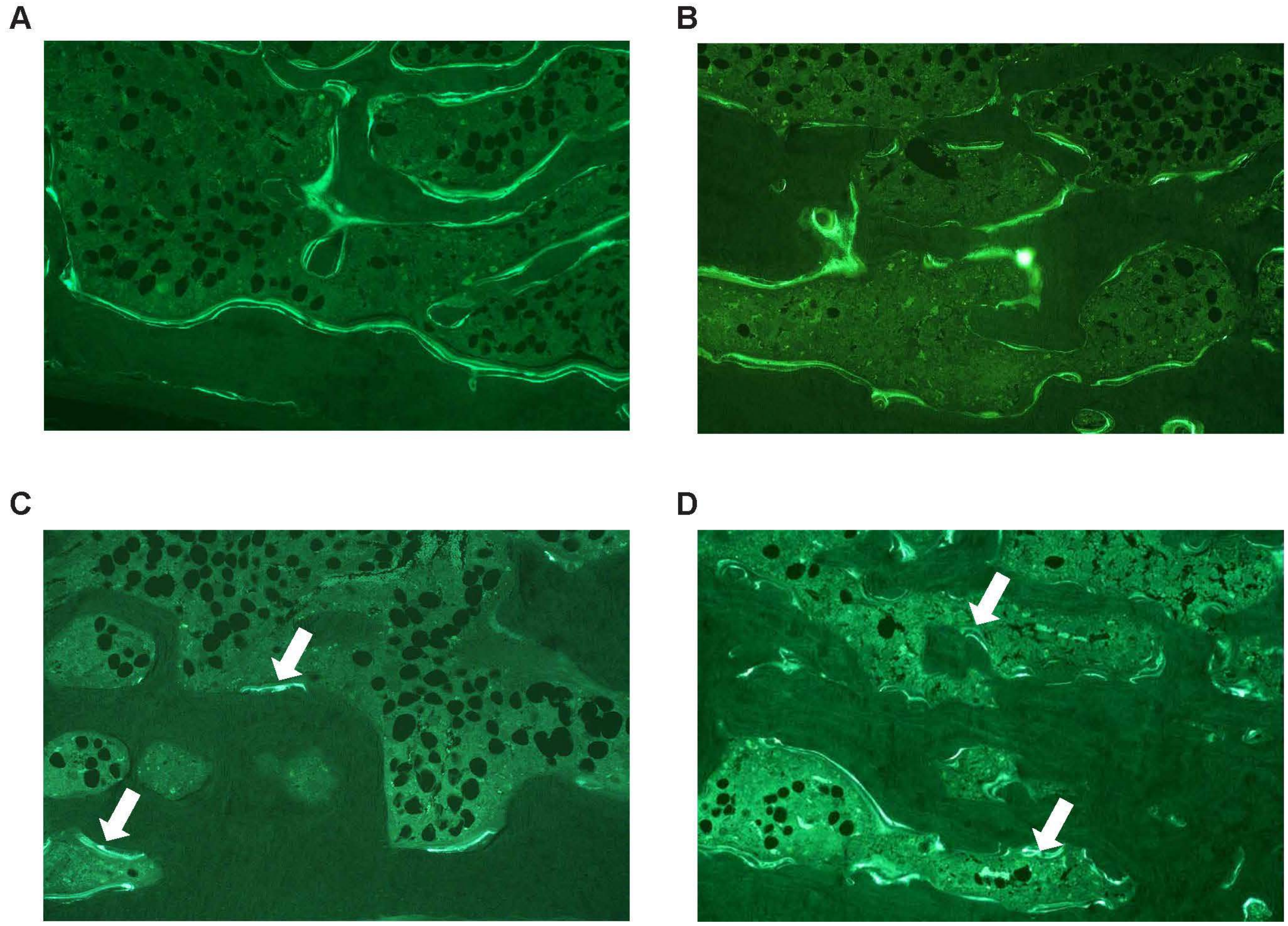

Bone surfaces in (A) group 1 (Veh/Veh/Scl-Ab) and (B) group 3 (OPG-Fc/ZOL/Scl-Ab) illustrating long smooth stretches of fluorochrome labeling (active modeling-based bone formation surfaces) on the Cn and Ec surfaces at week 20. Rare mini-modeling site (arrows) characterized by protrusions of focal areas of modeling-based bone formation into the marrow on Cn surfacesand Ec surfaces in (C) group 4 (Aln/OPG-Fc/Scl-Ab) at week 20. Increased mini-modeling (arrows) observed in (D) group 2 (OPG-Fc/OPG-Fc /Scl-Ab) at week 24. (Original magnification 40x).

Aln, alendronate; Cn, cancellous; Ec, endocortical; MBBF, modeling-based bone formation; Scl-Ab, sclerostin antibody; Veh, vehicle; ZOL, zoledronic acid.
